# The collection of Bathynellacea specimens of MNCN (CSIC) Madrid: microscope slices and DNA extract

**DOI:** 10.3897/zookeys.678.11543

**Published:** 2017-06-06

**Authors:** Ana I. Camacho, Beatriz A. Dorda, Begoña Sánchez Chillón, Isabel Rey

**Affiliations:** 1 Department of Biodiversity and Evolutionary Biology, Museo Nacional de Ciencias Naturales, CSIC, c/José Gutiérrez Abascal nº2, 28006, Madrid, Spain; 2 Department of Collection, Museo Nacional de Ciencias Naturales, CSIC, c/José Gutiérrez Abascal nº2, 28006, Madrid, Spain

**Keywords:** Bathynellacea, collections, Crustacea, DNA extract, groundwater fauna, invertebrate, MNCN (CSIC), permanent preparations, type collection

## Abstract

This is the first published database of a Bathynellacea Chappuis, 1915 collection of slices and DNA extracts. It includes all data of bathynellaceans (Crustacea: Syncarida) collected in the last 48 years (1968 to 2016) on the Iberian Peninsula and Balearic Islands, studied since 1984. It also includes specimens studied across many countries of Europe (Portugal, Romania, France, Italy, Slovenia, Bulgaria, and England), as well as some specimens obtained from samples of North America (Montana, Washington, Alaska and Texas), South America (Brazil, Chile and Argentina), Asia (China, Thailand, Vietnam, Mongolia and India), Africa (Morocco and Chad) and Australia (New South Wales –NSW- and Queensland). The samples come from groundwater (caves, springs, wells and hyporrheic habitat associated with rivers) obtained from both, sampling campaigns and occasional sampling efforts.

The data set includes 3399 records (2657 slices and 742 DNA extracts) corresponding to three families (Parabathynellidae Noodt, 1965, Leptobathynellidae Noodt, 1965 and Bathynellidae Grobben, 1905) of the order Bathynellacea; *the existence of three families is accepted, but this is a controversial issue and here is not the appropriate context to address this problem*; 52 genera and 92 species formally described, in addition to 30 taxa under study and, thus, still unpublished. This represents more than half of all the genera known worldwide (80) and almost one third of the species currently known in the world (329, which increases every year).

This dataset contains especially relevant collection that includes holotypes and type series of 43 new species of Bathynellacea (33 from the Parabathynellidae and ten from the Bathynellidae) described by Ana I. Camacho (AIC hereinafter); eleven of these are the type species for new genera described from all around the world, ten belonging to the Parabathynellidae and one from the Bathynellidae. As previously mentioned, these new species come from all continents, although 26 of them are from the Iberian Peninsula.

The most important feature of this collection is that it has been created and reviewed by a specialist of the group (AIC), and each specimen, regardless of its shape (either permanent slices or DNA extracts), includes taxonomic, geographical and authorship information. The specialist has been involved in all stages of the process, from field sampling to the digitization of the results we are now presenting, and has worked in close collaboration with the curators responsible for the different collections involved in this project.

## General description

**Figure 1.**
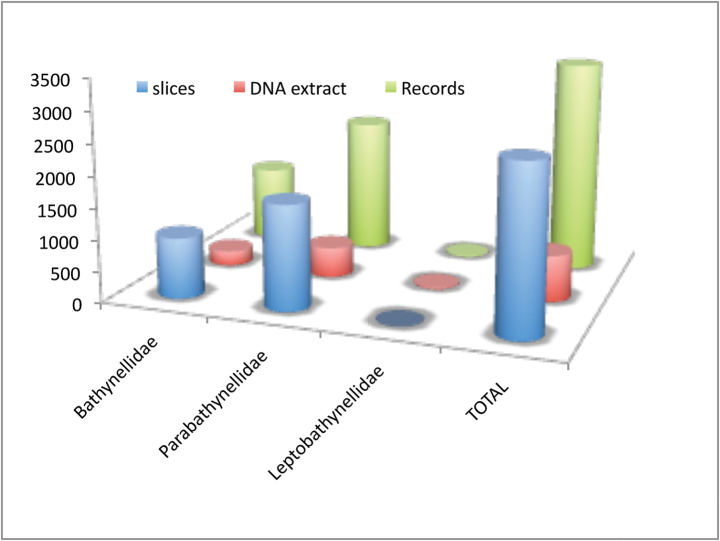
Familiy records in the MNCN collections.

**Figure 2.**
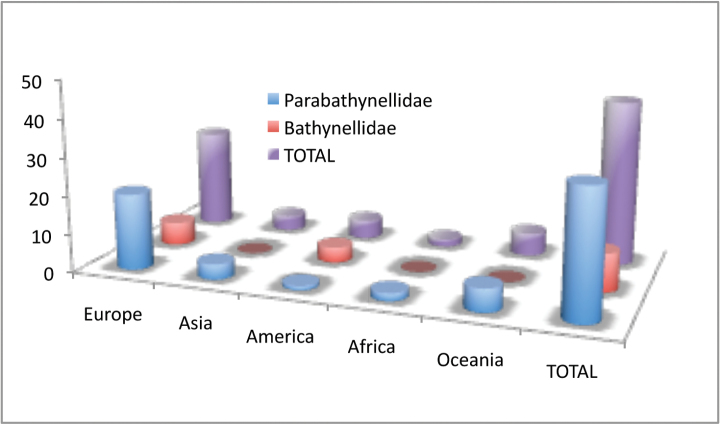
Bathynellacea holotypes by families and continents in the MNCN collections.

**Figure 3.**
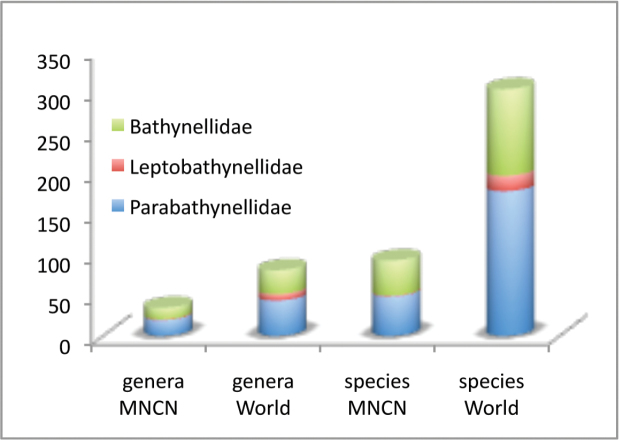
Bathynellacea genera by families in the MNCN collections versus world.

**Figure 4.**
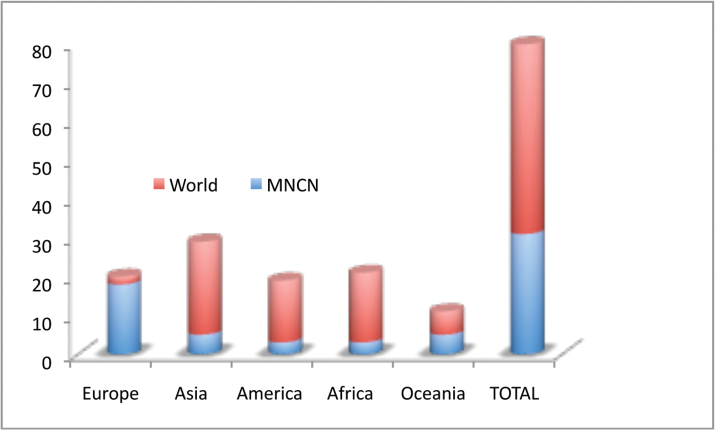
Number of genera of Bathynellacea by continents present in the MNCN collections versus world.

**Purpose**: The collections of the MNCN in Madrid hold the largest collection of Crustacea Bathynellacea in the world, with 3399 records (Figure 1) corresponding to 2657 permanent slices and 742 DNA extracts and their relevant taxonomic, geographical, and authorship information. From these, 2169 records (1683 permanent slices and 486 DNA extracts) belong to the Parabathynellidae, 1211 (974 permanent slices and 237 DNA extracts) belong to the Bathynellidae, and 20 (all DNA extracts) to the Leptobathynellidae (Figure 1). The objective of this work is to highlight the value of this collection by presenting it to the researcher community. Its importance is not only due to the number of specimens, but also due to their representativeness both taxonomically and spatially. What is also important is the number of types and type series it includes (holotypes and type series of 43 species coming from all continents) (Figure 2) and in their state of preservation which ensures its future utility. There are specimens from 31 different genera, from the 80 in total that are recognized worldwide (Figure 3), which belong to the three families currently known. This adds up to almost one third of all the species known in the world (94 of the 329 species formally described) (Table 1) (Figure 4). The collection includes specimens from all continents, from populations in Alaska to the South of Australia, although there is a predominance of European species, particularly from the Iberian Peninsula.

**Table 1.** Present taxa (families and genera) and species number from these genera in the collections of the MNCN and in the world by continent. % world representation in this database. *Oceania= Geopolitic region (Australia and New Zealand in this paper). ** Total number of world species is approximate, because there are new species in study and “in press”, and the number change every year.

Taxa	Species Number (MNCN collection/continent)					TOTAL species	**TOTAL species	Species number with DNA extract	Indeterminated species number/with DNA extract
	Europe	Asia	America	Africa	*Oceania	MNCN (%)	World	Collection MNCN	Collection MNCN
**Parabathynellidae**	**33/41**	**4/73**	**3/19**	**3/23**	**7/51**	**50 (28.2)**	**207**	**26**	**17/9**
***Iberobathynella***	21/22	0/0	0/0	0/0	0/0	21 (95.4)	22	14	6/6
***Paraiberobathynella***	2/2	0/0	0/0	1/1	0/0	3 (100)	3	3	2/2
***Guadalopebathynella***	1/1	0/0	0/0	0/0	0/0	1 (100)	1	1	0/0
***Hexaiberobathynella***	2/2	0/0	0/0	0/0	0/0	2 (100)	2	2	0/0
***Parabathynella***	2/3	0/0	0/0	0/0	0/0	2 (66.6)	3	0	1/0
***Hexabathynella***	5/11	0/0	1/6	0/3	1/3	7 (30.4)	23	3	3/0
***Paraeobathynella***	0/0	1/1	0/0	0/0	0/0	1 (100)	1	0	0/0
***Skethinella***	0/0	1/1	0/0	0/0	0/0	1 (100)	1	0	0/0
***Sinobathynella***	0/0	1/1	0/0	0/0	0/0	1 (100)	1	0	0/0
***Siambathynella***	0/0	1/1	0/0	0/0	0/0	1 (100)	1	1	0/0
***Montanabathynella***	0/0	0/0	1/1	0/0	0/0	1 (100)	1	0	0/0
***Octobathynella***	0/0	0/0	0/0	0/0	1/1	1 (100)	1	0	0/0
***Notobathynella***	0/0	0/0	0/0	0/1	2/8	2 (22.2)	9	0	2/0
***Chilibathynella***	0/0	0/1	0/1	0/0	2/3	2 (40)	5	0	2/0
***Onychobathynella***	0/0	0/0	0/0	0/0	1/1	1 (100)	1	0	0/0
***Haplophallonella***	0/0	0/0	0/0	1/2	0/0	1 (50)	2	1	0/0
***Racovitzaibathynella***	0/0	0/0	0/0	1/3	0/0	1 (33.3)	3	1	0/0
***Texanobathynella***	0/0	0/0	1/2	0/0	0/0	1 (50)	2	0	1/1
**Leptobathynellidae**	**0/0**	**1/4**	**0/10**	**0/5**	**0/0**	**1(5)**	**19**	**1**	**0**
*Parvulobathynella*	0/0	1/3	0/3	0/2	0/0	1 (12.5)	8	1	0
Bathynellidae	33/51	1/33	6/13	0/5	1/1	43 (40.6)	103	10	16+?/13
*Vejdovskybathynella*	5/7	0	0	0	0	5 (71.4)	7	3	3/3
*Pacificabathynella*	0/0	0	4/5	0	0	4 (80)	5	1	1/0
*Paradoxiclamousella*	2/2	0	0	0	0	2 (100)	2	2	3/3
*Clamousella*	1/1	0	0	0	0	1 (100)	1	0	3/3
*Gallobathynella*	3/4	0	0	0	0	5 (71,4)	7	3	2/2
*Meridiobathynella*	2/2	0	0	0	0	2 (100)	2	0	2/0
*Bathynella*	15/29?	1/16	2/5	0	1/1	19 (38?)	51?	0	2/2
*Delamareibathynella*	1/1	0	0	0	0	1 (33.3)	3	0	0/0
*Pseudobathynella*	1/2	0	0	0	0	1 (50)	2	0	0/0
*Sardobathynella*	1/1	0	0	0	0	1 (100)	1	0	0/0
*Vandelibathynella*	1/1	0	0	0	0	1 (100)	1	0	0/0
*Antrobathynella*	1/1	0/1	0	0	0	1 (50)	2	1	0/0
Total Bathynellacea	66/92	6/110	9/42	3/33	8/52	94 (30)	329	37	33+?/22

**Figure 5.**
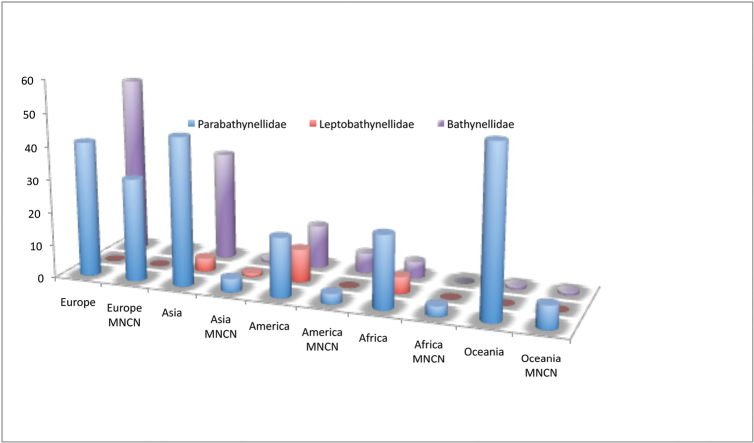
Number of species by families and continents present in the MNCN collections versus world.

This particular group of crustaceans is slowly showing the true magnitude of its diversity, and the collection presented here is a proof of this. It was traditionally considered a rare group with very low diversity mainly due to the fact that its habitat (groundwater) is rarely sampled, and that its presence and density is on average low. This, together with the difficulty for humans to access its environment, as well as the complex and time-consuming taxonomic research the group implies due to the small size of the species (most of the species are not larger than a millimeter) and their morphological complexity of their numerous appendices (e.g., thoracopod VIII male transformed into a copulatory organ), has prevented many researchers devoting their time to their study over the years. Nevertheless, one of the authors (AIC) has devoted over 30 years of work to produce the collection we are presenting here. We are convinced that the relevance of the collection is already reason enough for its publication, especially due to the important information on the Iberian Peninsula and Balearic Islands, which is currently one of the best-studied regions in terms of bathynellaceans, and linked with this effort, also the region with the highest diversity of this group of crustaceans in the world (Camacho et al., 2014). There are 58 species known for this particular region, 41 formally described, and at least 17 more that have been identified as new species, but are pending description. This includes many cryptic species identified thanks to molecular studies (Camacho et al., 2011, 2012, 2013a, b). All of the above are represented through permanent slices in the collection we present here, plus DNA extracts of 41 of the species, although currently not all of them include the gene sequences. In addition to all of these, the collection also includes many other European species (66), as well as species from Asia (6), America (9), Australia (8) and Africa (3) (see Table 1 and Figure 5).

The present paper is an important contribution that offers basic and rigorous taxonomic information, which is updated and can be potentially useful for subterranean biodiversity studies (identifying hotspots), and also for ecology and conservation studies, particularly for estimating future global changes as the specimens recorded range from 1986 to the present.

Our aims for publishing this dataset are 1) describing the Bathynellacea collection of permanent slices and DNA extract of the MNCN, 2) show the first data set of holotype and type series collection of Bathynellacea in the world, 3) providing information on the diversity and distribution of groundwater fauna in the world and 4) offering the first dataset of Bathynellacea permanent slices in the world to the scientific community in the hopes of promoting other researchers to publish their different groundwater fauna datasets.

### Additional information

Table 1 shows the present taxa (families, genera and species) in the collections of the MNCN and in the world by continent with % representation in the collections.

Table 2 includes information on all the new species of Bathynellacea described by authors, including the catalogue number of holotype and DNA voucher from specimens of type localities (where available) from classic Crustacea and “Tissue and DNA” collections of the MNCN and the numbers of specimens of type series.

**Table 2.** List of species of Bathynellacea with holotypes and type series deposited in the collections (Arthropods and Tissues and DNA) of the Museo Nacional de Ciencias Naturales de Madrid (CSIC) (Spain). (H) Hyporheic habitat, gravel bank of rivers; (*) Genus described by author(s) of this paper. (**) The holotype and type series of new species described from Spain not deposited in MNCN.

Taxa	Habitat	Type locality	Province	Country	Description year	Type serie male/female	Holotype voucher MNCN 20.04/	Loc. type voucher MNCN:ADN:
**Parabathynellidae**								
***Iberobathynella***								
*I. imuniensis*	Cave	Torca Morteros	Burgos	Spain	1987	10/4	4642	29146-29446
*I. rouchi*	River (H)	Guadalope	Teruel	Spain	1987	5/4	4641	
*I. ortizi*	Cave	Rei Cintolo	Lugo	Spain	1989	4/5	4643	54609-54622
*I. cantabriensis*	Cave	El Calderón	Cantabria	Spain	1998	5/3	4639	
**I. magna	Cave	Del Infierno	Asturias	Spain	1998	**	–	
*I. parasturiensis*	Cave	Treslajorá, CO.209	Asturias	Spain	1998	7/2	4640	
*I. paragracilipes*	Well	Quejigo	Huelva	Spain	1998	8/10	4638	
*I. celiana*	River (H)	Arroyo Torrecilla	Sevilla	Spain	2003	0/1	5323	29452
*I. serbani*	River (H)	Lima	–	Portugal	2003	1/3	5321	
*I. pedroi*	River (H)	Mondego	Coimbra	Portugal	2003	1/5	5320	
*I. guarenensis*	Cave	Erizo, Ojo Guareña	Burgos	Spain	2003	0/4	5322	
*I. lamasonensis*	Cave	Estragüeña	Cantabria	Spain	2005	5/7	5911	
*I. cornejoensis*	Cave	Redonda	Burgos	Spain	2005	5/2	5912	29946-29952
*I. burgalensis*	Cave	Ojo Guareña, OG53	Burgos	Spain	2005	5/3	6063	29220-29542
*I. andalusica*	Well	Fuentes Andalucia	Sevilla	Spain	2007	3/8	7966	29418-29438
*Paraiberobathynella								
*Pi. notenboomi*	Well	Orihuela	Alicante	Spain	1989	4/4	4644	
*Guadalopebathynella								
*G. puchi*	River (H)	Guadalope	Teruel	Spain	1998	14/12	4450	
*Hexaiberobathynella								
*Hi. hortezuelensis*	Well	Hortezuella	Soria	Spain	1998	10/10	4451	
***Hexabathynella***								
*H. nicoleiana*	River (H)	Jarama	Madrid	Spain	1986	10/14	4645	
*H. valdecasasi*	River (H)	Torcón	Guadalajara	Spain	2003	1/2	4866	
*H. sevillaensis*	Cave	Santiago Grande	Sevilla	Spain	2005	8/7	5913	29545-29565
*Paraeobathynella								
*P. vietnamensis*	Cave	Hang Trinh	Dao Bo Hon	Vietnam	2005	15/13	5911	
*Skethinella								
*S. trontelji*	Cave	Hon Rom	Vinh Ha Long	Vietnam	2005	3/0	5912b	
*Sinobathynella								
*S. decamera*	Cave	Si Haizi	Dens	China	2006	1/1	7048	
*Siambathynella								
*S. laorsiae*	Cave	Tham Yai Nam	Phetchabun	Thailand	2011	6/3	8568	
*Montanabathynella								
*M. salish*	River (H)	Junko	Montana	USA	2009	1/1	7970	
*Octobathynella								
*O. peelensis*	Well	Tamworth	NSW	Australia	2011	1/3	8226	
***Notobathynella***								
*N. octocamura*	Well	Bundaberg	Queensland	Australia	2011	2/4	8229	
*N. pentatrichion*	Well	Bundaberg	Queensland	Australia	2011	4/2	8232	
***Chilibathynella***								
*C. joshuai*	Well	Dubbo	NSW	Australia	2011	3/1	8558	
*C. digitus*	Well	Tamworth	NSW	Australia	2011	3/3	8561	
*Onychobathynella								
*O. bifurcata*	Well	Hunter	NSW	Australia	2011	0/3	8564	
***Haplophallonella***								
*H. irenae*	River (H)	Uet Duar	Toutous	Chad	2016	16/9	10148	29986-29987
***Racovitzaibathynella***								
*R. dumonti*	River (H)	Uet Duar	Toutous	Chad	2016	16/9	10150	29981-29988
**Bathynellidae**								
***Vejdovskybathynella***								
*V. edelweiss*	Cave	Ojo Guareña, OG16	Burgos	Spain	2007	11/20	7791	29414-29482
*V. caroloi*	Cave	Molino, Matienzo	Cantabria	Spain	2007	5/10	7792	
*V. pascalis*	Cave	Cubilla, Ogarrio	Cantabria	Spain	2007	1/1	7793	
*V. vasconica*	Cave	Goikoetxe	Vizcaya	Spain	2013	7/18	9119	29623-29889
***Pacificabathynella***								
*P. kalispellensis*	Well	Flathead County	Montana	USA	2009	3/3	8090	
*P. stanfordi*	Well	Graham Channel	Montana	USA	2009	4/7	8093	
*P. ruthae*	Well	Flathead County	Montana	USA	2009	6/4	8096	
*P. yupik*	River (H)	Kwethluk	Alaska	USA	2015	3/21	10092	29963-29967
*Paradoxiclamousella								
*P. fideli*	Cave	Pozo Agua, CO69	Asturias	Spain	2013	6/14	8855	29746-29753
*P. pirata*	Cave	Río Chico	Cantabria	Spain	2013	2/5	8877	29998-29999

Table 3 is a short list of species and localities of Bathynellacea of which there are DNA extracts in the collection of the MNCN.

**Table 3.** List of species and localities of Bathynellacea with extracts of DNA in the collection of the MNCN.

Taxa	Habitat	Type locality	Province	Country	Voucher ADN/
**Parabathynellidae**					
***Iberobathynella***					
*I. andalusica*	Well	Fuentes Andalucia	Sevilla	Spain	29418-29438
*I. asturiensis*	Cave	Pruneda	Asturias	Spain	29190-29828
*I. asturiensis*	Cave	Tresavarilla	Cantabria	Spain	29192-29826
*I. burgalensis*	Cave	Ojo Guareña, OG53	Burgos	Spain	29220-29542
*I. cantabriensis*	Cave	Calderón, CO.099	Cantabria	Spain	29376-29838
*I. cantabriensis*	Cave	Pelacristo, CO261	Asturias	Spain	29148-29492
*I. cantabriensis*	Cave	Treslajorá, CO.209	Asturias	Spain	29295-29571
*I. cantabriensis*	Cave	Lobos, CO.276	Cantabria	Spain	29537-29798
*I. cavadoensis*	River (H)	Cavado River		Portugal	29183-29840
*I. cavadoensis*	River (H)	Tamuxo stream	Pontevedra	Spain	29234-29832
*I. celiana*	River (H)	Viar stream	Sevilla	Spain	29452
*I. cornejoensis*	Cave	Redonda	Burgos	Spain	29946-29952
*I. imuniensis*	Cave	Torca Morteros	Burgos	Spain	29146-29446
*I. imuniensis*	Cave	Bernías	Burgos	Spain	29776-29792
*I. imuniensis*	Cave	Lunada	Burgos	Spain	29989-29994
*I. imuniensis*	Cave	V-142	Burgos	Spain	54559-54564
I. cf imuniensis	Cave	El Becerral	Cantabria	Spain	54569
I. cf imuniensis	Cave	Fonda	Vizcaya	Spain	54658-54663
*I. lusitanica*	River (H)	Cavado River		Portugal	29184-29842
*I. magna*	Cave	Helechosa, CO	Cantabria	Spain	29939
*I. magna*	Cave	Treslajorá, CO.209	Asturias	Spain	29294-29575
*I. magna*	Cave	Pelacristo, CO.261	Asturias	Spain	29367-29494
*I. ortizi*	Cave	Rei Cintolo	Lugo	Spain	54609-54622
*I. paragracilipes*	Well	Quejigo, Jabugo	Huelva	Spain	29821-29248
*I. paragracilipes*	Well	Ermita San Isidro	Huelva	Spain	29209
*I. parasturiensis*	Cave	Treslajorá, CO.209	Asturias	Spain	29553-29589
*I. parasturiensis*	Cave	La Nava, CO.044	Cantabria	Spain	29609-29916
*I. parasturiensis*	Cave	Divisada, CO.275	Asturias	Spain	29193-29312
*I. rouchi*	River (H)	Guadalope River	Teruel	Spain	29174-29238
*I. rouchi*	River (H)	Cinca River	Huesca	Spain	29178-29213
*I. rouchi*	River (H)	Alfambra River	Teruel	Spain	29176-29254
*I.* sp	River (H)	Mondego River	Coimbra	Portugal	29868
*I.* sp	Cave	Treslajorá, CO.209	Asturias	Spain	29587-54558
*I.* sp	Cave	Pozo Agua, CO.069	Asturias	Spain	29704-29738
*I.* sp	Cave	Torca Tejo, CO.246	Asturias	Spain	29264-29831
*I.* sp	Cave	Grañaja, CO.150	Cantabria	Spain	29290-29830
*I.* sp	Cave	del Pilar, CO.314	Asturias	Spain	29168-54547
*I.* sp	Cave	Si 44	Alava	Spain	29219-29616
*I.* sp	Cave	San Juan	Vizcaya	Spain	29968
*I.* sp	Cave	Soplao Mina Elvira	Vizcaya	Spain	29969-29974
*I.* sp	Cave	Astui	Vizcaya	Spain	29978-29980
*I.* sp	Cave	Lamiñas	Vizcaya	Spain	29975-29977
*I.* sp	Cave	Monasterio (CO231)	Asturias	Spain	29300
*I.* sp 1	Cave	del Pilar, CO.314	Asturias	Spain	9001-29759
*I.* sp 1	Cave	Lobos, CO.276	Cantabria	Spain	29538-29539
*I.* sp 2	Cave	Treslajorá, CO.209	Asturias	Spain	29559-29658
*I.* sp 2	Cave	del Pilar, CO.314	Asturias	Spain	29472-29756
*I.* sp 2	Cave	Carnero, CO.220	Cantabria	Spain	29734
*I.* sp 3	Cave	Pozo Agua, CO.069	Asturias	Spain	29705-54542
*I.* sp 3	Cave	del Pilar, CO.314	Asturias	Spain	29473
*I.* sp 4	Cave	Los Orios, CO.089	Asturias	Spain	29488
*Paraiberobathynella					
Pi. cf fagei	Cave	Sima La Higuera	Murcia	Spain	29665-54552
Pi. cf fagei	Cave	La Pileta	Málaga	Spain	54581-54591
Pi. cf fagei	River (H)	Jucar River	Valencia	Spain	54663-54636
Pi. cf fagei	River (H)	Vélez River	Málaga	Spain	29819-29820
Pi. cf fagei	River (H)	Turia River	Valencia	Spain	54566-54567
Pi. cf fagei	River (H)	Alcanadre River	Huesca	Spain	29929
Pi. cf maghrebensis	Well	Nador-Bercame	Maghreb	Morocco	29931-29962
*Pi. fagei*	Cave	Campanet	Mallorca	Spain	29200
*Pi. fagei*	Cave	Génova	Mallorca	Spain	29660
*Pi. fagei*	Cave	Son Berenguer	Mallorca	Spain	29292-29293
*Pi. fagei*	Cave	Sa Bassa Blanca	Mallorca	Spain	29194-29928
*Pi. fagei*	River (H)	Areta River	Navarra	Spain	29180-29818
*Pi. fagei*	River (H)	Ter River	Gerona	Spain	29475
*Pi. fagei*	Well	Los Picos	Valencia	Spain	29221-29802
*Pi. fagei*	River (H)	Lima River		Portugal	29805-29806
*Pi. fagei*	River (H)	Esla River	León	Spain	29807-29808
*Pi. fagei*	River (H)	Orza River	León	Spain	29182
*Pi. fagei*	River (H)	Sella River	Asturias	Spain	29235-29812
*Pi. fagei*	Well	German	Almería	Spain	29297-29800
*Pi. fagei*	River (H)	Frio stream	Granada	Spain	29809-29810
*Pi. fagei*	River (H)	Lucainena stream	Granada	Spain	29181-29816
*Pi. fagei*	River (H)	Alfambra River	Teruel	Spain	29803
*Pi. notemboomi*	Well	Los Picos	Valencia	Spain	29189
*Pi.* sp	Well	Navas de Riofrío	Segovia	Spain	29661
***Hexaiberobathynella***					
*Hi. hortezuelensis*	Well	Hortezuella	Soria	Spain	29186-29851
*Hi. mateusi*	River (H)	Jarama	Madrid	Spain	29187-29847
*Guadalopebathynella*					
*G. puchi*	River (H)	Guadalope	Teruel	Spain	29177-29260
*H. minuta*	River (H)	Pinhao	Balsa	Portugal	29261
*H. minuta*	River (H)	Rivera de Huelva	Sevilla	Spain	29173
*H. nicoleiana*	River (H)	Jarama	Madrid	Spain	29231-29845
*H. sevillaensis*	Cave	Santiago Grande	Sevilla	Spain	29545-29565
***Haplophallonella***					
*H. irenae*	River (H)	Uet Duar	Toutous	Chad	29986-29987
***Racovitzaibathynella***					
*R. dumonti*	River (H)	Uet Duar	Toutous	Chad	29981-29988
*Siambathynella*					
*S. laorsriae*	Cave	Tham Yai	Phetchabum	Thailand	29617-29549
*Texanobathynella*					
*T.* sp	River (H)	Live Oak creek	Texas	USA	54641-56646
**Bathynellidae**					
***Vejdovskybathynella***					
*V. caroloi*	Cave	Gándara	Cantabria	Spain	29978-29900
*V. edelweiss*	Cave	Ojo Guareña, OG09	Burgos	Spain	29415-29482
*V. edelweiss*	Cave	Ojo Guareña, OG01	Burgos	Spain	29471-29483
*V. edelweiss*	Cave	Ojo Guareña, OG16	Burgos	Spain	29414
*V. edelweiss*	Cave	La Mina	Burgos	Spain	29945
*V. edelweiss*	Cave	Racino	Burgos	Spain	29953-29958
*V. edelweiss*	Cave	Huesos	Burgos	Spain	29440-29450
*V. vasconica*	Cave	Goikoetxe	Vizcaya	Spain	29623-29889
*V.* sp 1	Cave	Ojo Guareña, Erizos	Burgos	Spain	29487
*V.* sp 1	Cave	Río Chico	Cantabria	Spain	294722-54632
*V.* sp 2	Cave	Redonda	Burgos	Spain	29523-29524
*V.* sp 2	Cave	Imunía	Burgos	Spain	29917-29918
***Pacificabathynella***					
*P. yupik*	River (H)	Kwethluk	Alaska	USA	29963-29967
***Paradoxiclamousella***					
*P. fideli*	Cave	Pozo Agua, CO069	Asturias	Spain	29746-29753
*P. fideli*	Cave	Fuente Carnero	Cantabria	Spain	29375-29735
*P. fideli*	Cave	Pilar, CO314	Asturias	Spain	29717-29718
P. cf fideli	Cave	Treslajorá, CO209	Asturias	Spain	29593-29596
P. cf fideli	Cave	La Nava, CO034	Asturias	Spain	29914-29915
*P. pirata*	Cave	Río Chico	Cantabria	Spain	29998-29999
*P.* sp1	River (H)	Alcanadre	Huesca	Spain	29286-29804
*P.* sp2	River (H)	Pinhao		Portugal	29283
*Gallobathynella*					
*G. boui*	Cave	Deveze	Courniou	France	54600-54601
*G. coiffaiti*	Cave	Falgas	Rieussec	France	54602-54603
*G. tarissei*	Cave	Limousis		France	54592-54593
*G.* sp	Cave	Les Perles	Melagues	France	54594-54595
*G.* sp	Cave	Lacombe	Camboumes	France	54596-54597
*G.* sp	Spring	Janoye-Figuier	Penne	France	54598-54599
*G.* sp1	River (H)	Jarama	Madrid	Spain	29307-29860
*Antrobathynella*					
*A. stammeri*	Cave	Ogof Draemen	South Wales	England	54647-54657
*Bathynella*?					
*B.*? sp		Edwards Aquifer	Texas	USA	29943-54640
*B.*? sp	River (H)	Guadiato	Córdoba	Spain	29622
Undeterminated genus	Cave	Menor	Asturias	Spain	29843
Undeterminated genus	Cave	Fuentemolinos	Burgos	Spain	29866-29867
Undeterminated genus	River (H)	Stream	Sevilla	Spain	29142-29453
*Clamousella* Unpublished					
*C.* sp 1	River (H)	Stream		Portugal	29204-29852
*C.* sp2	River (H)	Pinhao Stream		Portugal	29282
*C.* sp3	River (H)	Stream	Valencia	Spain	29288-29289
**Leptobathynellidae**					
*Parvulobathynella*					
*P. distincta*	River (I)	Godavari	Andhra Prades	India	29683-29942

Section 1 of the bibliography includes a list of the publications citing the bathynellaceans included in this dataset.

## Project details

**Project title**: Data Base of Bathynellacea specimens collection of MNCN (CSIC) Madrid: microscope slices (permanent slices) and DNA extracts.

**Personnel digitization**: Camacho AI

**Determination specialist**: Camacho AI

**Administrative contact**: Dorda BA

**Bathynellacea determination specialist**: Camacho AI

**Funding**: Fauna Ibérica I (DGICYT PB87-0397); Fauna Ibérica II (DGICYT PB89-0081); Fauna Ibérica III (DGICYT PB92-0089); Inferencia de Patrones Biogeográficos a pequeña escala (DGICYT PB96-0894); Inventario y Catalogación informática de la Biodiversidad acuática subterránea de la Península Ibérica, Baleares y Macaronesia (CICYT REN2000-2004 GLO); Protocols for the Assessment and Conservation of aquatic life in the subsurface (PASCALIS), European Union Proposal EVK2-2001-00086 (Contract: EVK2- CT-2001-00121); Biodiversidad Faunística en el sector turístico del Complejo Ojo Guareña: Evaluación de la Influencia de la presión humana en algunas de sus poblaciones de invertebrados (Contract CSIC- Junta de Castilla León, 2002-2004); Sobre el origen y distribución de la fauna acuática subterránea (CICYT CGL2005-02217/BOS); Colonización, Éxito Evolutivo y Biodiversidad Faunística del Complejo Kárstico de Ojo Guareña” En el Monumento Natural de Ojo Guareña (Burgos) (Contract CSIC- Junta de Castillay León, 2006-2009); Estudio piloto para la detección a diferentes escalas geográficas de procesos evolutivos relacionados con el origen de la biodiversidad en grupos de invertebrados singulares (MICINN CGL2010-15786, subprograma BOS; Identificación de especies crípticas mediante análisis filogeográficos y filogenias multigénicas: una revisión de la diversidad real en grupos taxonómicamente complejos (MINECO CGL2015-66571-P/ FEDER).

**Study area descriptions/descriptor**: The area of study includes the whole world. There are over 200 sites from the Iberian Peninsula and Balearic Islands (Camacho et al., 2014), as well as other European localities from France, Italy, Slovenia, Bulgaria, Rumania and England. In the case of the American continent, the collection includes specimens from a locality in Brazil, another one in Chile, and one more in Argentina, together with several localities across the USA: Texas, Montana, Washington and Alaska. The Asian specimens were collected in several caves in China, Vietnam, Thailand, and some localities in South India. The specimens from Australia are from New South Wales and Queensland. The African samples come from two localities in Morocco and one in Chad.

Several sampling dates ranging from 1968 to 2016.

The samples come from groundwater caves, springs, wells and interstitial environment (hyporheic) of the epigean river where the stygobionts fauna living in them can be collected.

**Design description**: This dataset was developed to contribute to the knowledge of a group of groundwater Crustacea, Bathynellacea, of worldwide distribution and sparse study; to identify endemic fauna at different geographic scales (country, counties and localities); to value this collection of Madrid MNCN and encourage other colleagues to show less striking results of their work. Prior to digitization, the taxonomic identification pre-existing was reviewed by the specialist AIC. The dataset is exported to Darwin Core v1.2 format and uploaded to the IPT of the GBIF Spanish node (http://www.gbif.es/ipt/resource?r=mncn-artp). Darwin Core elements included in the dataset structure are listed in the dataset description section.

**Data published through** GBIF: http://www.gbif.es/ipt/resource?r=mncn-artp; http://www.gbif.org/dataset/07f0789f-c777-4c99-acb3-815c78c7db81

## Taxonomic coverage

**Figure 6.**
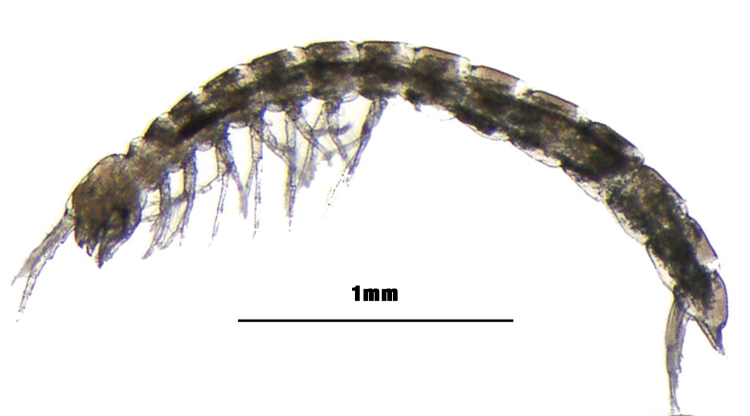
Habitus of Parabathynellidae family: Paraiberobathynella cf. fagei (Delamare Deboutteville & Angelier, 1950) from Higuera cave, Murcia (Spain). Lateral view.

**General taxonomic coverage description**: This is a collection of slices and DNA extracts of Bathynellacea, a group of Crustacea Malacostraca (Figure 6) containing specimens from all known species for Spain, and high percentages of all species known in Europe, as well as some of those described in recent years (2006 onwards) in the other continents (Tables 1, 2 and 3). The collection includes all the samples obtained in the Iberian Peninsula and Balearic Islands since 1983 by AIC, also donated material from these areas and from different parts of the world to AIC for study, as detailed above. Most of the collection is identified to species level. The specimens still without identification to species level have been identified to genus or family level.

**Figure 7.**
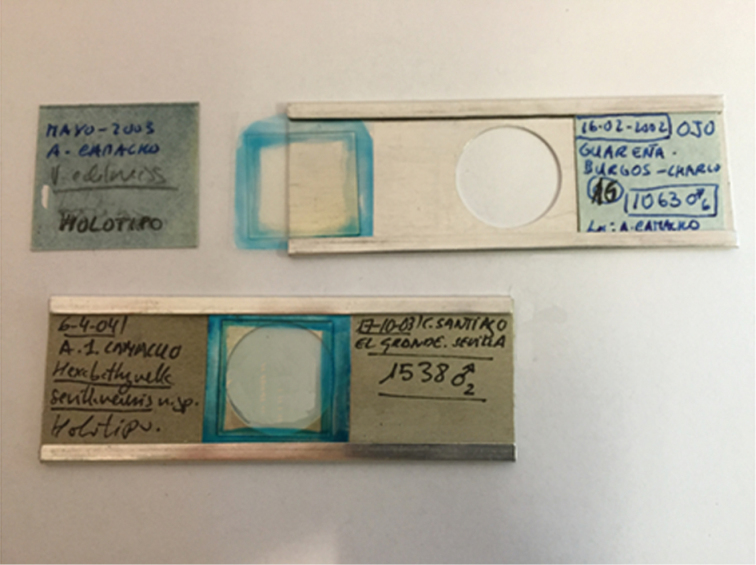
Permanent slides (special metal slides) of holotypes of the MNCN collections. Mounting medium: glycerine gelatin stained with methylene blue.

The three families of the order Bathynellacea: Bathynellidae, Parabathynellidae and Leptobathynellidae, are all represented in the collection, and in the case of the first two, in the shape of both DNA extracts and permanent slices (Table 3, Figs 1, 3, 7). Leptobathynellidae has been found in North America and southern hemisphere (Asia, Africa and South America) and includes 8 genera and 19 species, while in the collection of the MNCN contains 20 specimens in the shape of DNA extracts, which belong to a species from southern India *Parvulobathynella distincta* Ranga Reddy et al., 2011 (Table 1).

**Figure 8.**
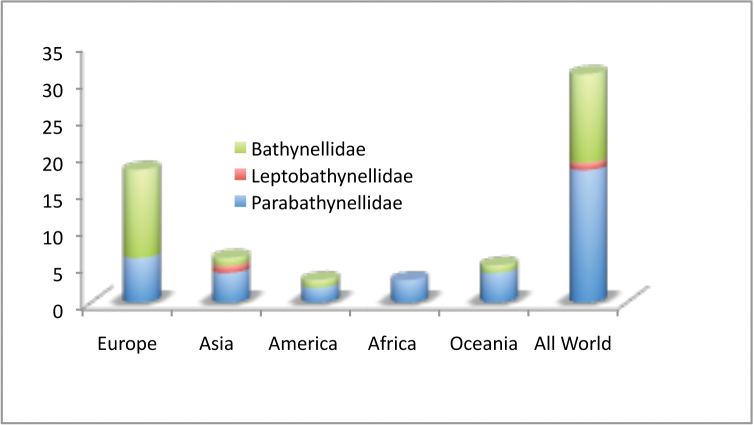
Number of genera of Bathynellacea by families and continents in the MNCN collections versus world.

All in all, of the 80 genera known worldwide, almost 40% (31 genera) are represented in the collection (Table 1). This is around 40% of the genera belonging to families Parabathynellidae (18 genera out of 43) and Bathynellidae (12 genera out of 29), and 13% of the genera from Leptobathynellidae (Figure 3). Europe is the continent with most representation in the collection, with 90% of the total genera known included (18 out of 20), followed by Australia with 45% of the genera (five out of 11). On the other hand, Africa remains with the lowest representation with only 14% of the known genera present in the collection (three out of 21). Asia (six out of 29) and America (four out of 19) are equally represented with 21% of the known genera included in the collection (Figure 4). Within the whole set of specimens included in the collection of the MNCN, the family Parabathynellidae has a higher number of genera included (18) when compared to Bathynellidae (12). Nevertheless if we only consider the European species, although the collection includes 100% of the Parabathynellidae species known (6), there are more species of Bathynellidae in total (11), due to their higher diversity. In the case of Africa, the collection does not include a single genus of the Bathynellidae family. In the case of America, Asia and Australia, only one genus is included (Figure 8).

The family Parabathynellidae includes approximately 207 species in total, and 50 of these are preserved in the collection (Tables 1, 2, 3). Out of these, more than half (27 species) are also represented by DNA extracts. There is also a high number of undetermined species, most with DNA extracts. The continent most widely represented in the collection is Europe with 100% of the know genera included, and over 75% (31) of all species known (41) (Figure 5). On the other hand, the least represented continent is Asia with hardly 9% of the known species included in the collection (four of 45 species). The rest of continents range between 13% and 17% of the species included in this collection. The genus *Iberobathynella* Schminke, 1973, endemic to the Iberian Peninsula and Balearic Islands, is the most diverse with 22 species, and also the most represented in the collection with 20 species. In addition, the collection of the MNCN also includes the 3 known species of the genus *Paraiberobathynella* Camacho & Serban, 1998, the 2 known species of de *Hexaiberobathynella* Camacho & Serban, 1998, and the only known species of the genus *Guadalopebathynella* Camacho & Serban, 1998. The genus *Parabathynella* Chappuis, 1926 has a total of three species in all of Europe, and two of them are included in the collection. Finally, the cosmopolitan genus *Hexabathynella* Schminke, 1972, which includes 23 species worldwide, is represented in the collection by six species, three of them including DNA extracts (Table 3).

The Leptobathynellidae, only known from North America and the Austral hemisphere in Asia, Africa and America with 19 species, is included in the collection through 20 specimens belonging to a single species.

**Figure 9.**
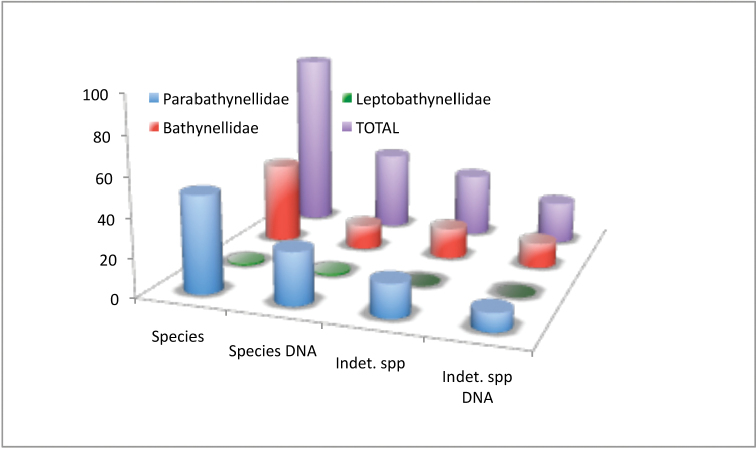
Number of species (total and DNA extract) of Bathynellacea by families in the MNCN collections.

**Figure 10.**
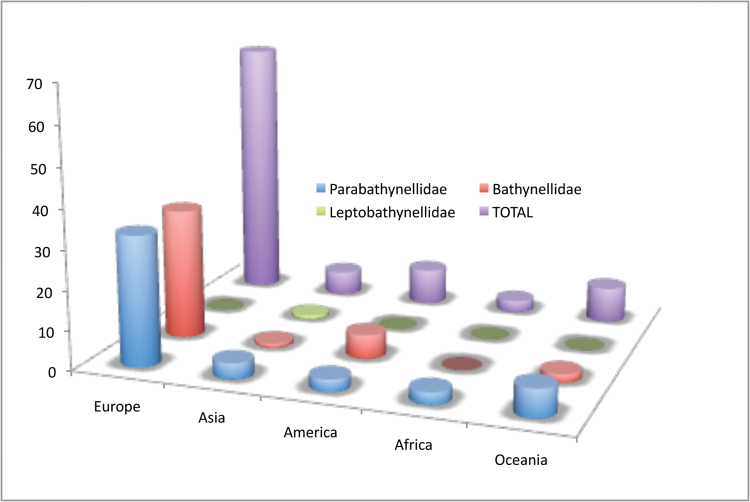
Number of species of Bathynellacea by continents and families in the MNCN collections.

The Bathynellidae is less known across the world than the Parabathynellidae, although particularly in Europe, where its generic and specific diversity is higher, it is the best known family, as well as the most represented in this collection, with 43 of the 103 known species worldwide included (approximately half of these are dubiously assigned to the genus *Bathynella* Vejdovsky, 1882, which some authors consider cosmopolite) (Figures 5, 9). In total, 13 of these species include DNA extracts in the collection (Table 3). There is also a high number of undetermined species, at least 16, and 13 of these include DNA extracts. The collection includes at least 35 European species in total (Table 1); 15 are assigned to the genus *Bathynella*, but should be revised based on the most recent discoveries offered by molecular techniques. The collection holds five of the seven species known for the genus *Gallobathynella* Serban et al., 1971, five of the seven species known from the genus *Vejdovskybathynella* Serban & Leclerc, 1984, and nine of the ten species assigned to the rest of European genera. There are DNA extracts in the collection of several of these. The presence of the genus *Pacificabathynella* Schminke & Noodt, 1988, in the collection is also important with 4 of the 5 American species known included. In the case of the species *P. yupik* Camacho et al., 2015 from Alaska, DNA extracts are also preserved. The rest of the continents have a relatively low representation (Figure 10).

**Figure 11.**
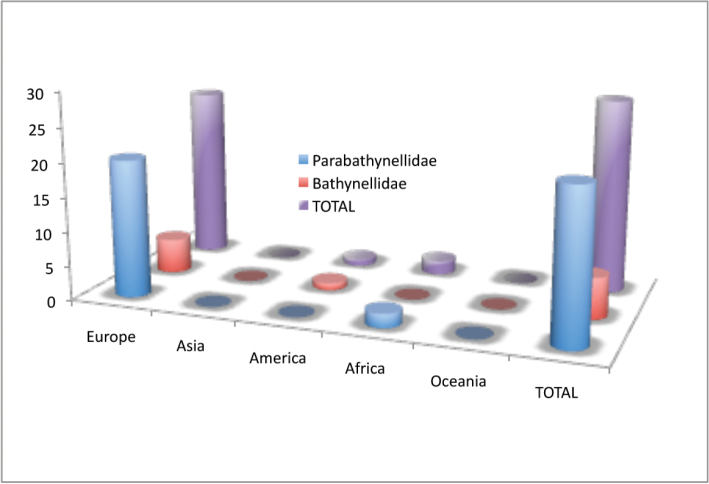
Number of holotypes (DNA extract) of Bathynellacea by continents and families in the MNCN collections.

It is worth noting the holotype collection and the type series of Bathynellacea housed at the MNCN. Table 2 contains a summary of the new taxa (11 genera and 43 species) described by AIC ranging across different families and continents, and whose holotypes and type series are deposited in the collections of the MNCN, either as permanent slices in the arthropod collection (Figure 9), or as DNA extracts in the tissue and DNA collection (Figures 2, 11). The Parabathynellidae includes 33 holotypes and the type series of ten genera coming from all continents: 20 holotypes come from Spain belonging to the genera *Iberobathynella*, *Guadalopebathynella*, *Paraiberobathynella*, *Hexaiberobathynella* and *Hexabathynella*. Four other holotypes belong to new genera and species from Thailand, China and Vietnam, another holotype is a new genus from Montana (USA), and other eight holotypes correspond to six Australian and two African species (Figure 2). In the case of the Bathynellidae, there are en holotypes, six Spanish species from two genera (*Paradoxiclamousella* Camacho et al., 2013a and *Vejdovskybathynella*), and 4 more from the USA (Montana and Alaska), all from the genus *Pacificabathynella* Schminke & Noodt, 1988. Table 4 includes all the details of these species and populations, including information on habitat, locality, year of description, the vouchers of the morphologic holotypes, as well as the molecular type series and the composition of the type series in terms of number of specimens. In the case of most of the newly described European species, from both families, as well as for the two African species and of *Pacificabathynella yupik* from Alaska, there are DNA extracts included in the collection (Figure 11).

**Table 4.** New taxa by families and continents of Bathynellacea with type series are deposited in the MNCN collection. * Oceania= Geopolitic region (Australia and New Zealand in this paper).

Taxa	Continent New genus /new species					TOTAL New genus/new species
	Europe	Asia	America	Africa	*Oceania	
**Parabathynellidae**	3/21	4/4	1/1	0/2	2/6	10/34
**Bathynellidae**	**1/6**	**0/0**	**0/4**	**0/0**	**0/0**	**1/10**
**Leptobathynellidae**	**0/0**	**0/0**	**0/0**	**0/0**	**0/0**	**0/0**
**Total Bathynellacea**	**4/26**	**4/4**	**1/5**	**0/2**	**2/6**	**11/44**

## Taxonomic ranks

Kingdom: Animalia

Phylum: Arthropoda

Class: Crustacea

Order: Bathynellacea

Family: Bathynellidae, Parabathynellidae, Leptobathynellidae.

Common names: does not exist

## Spatial coverage

**Figure 12.**
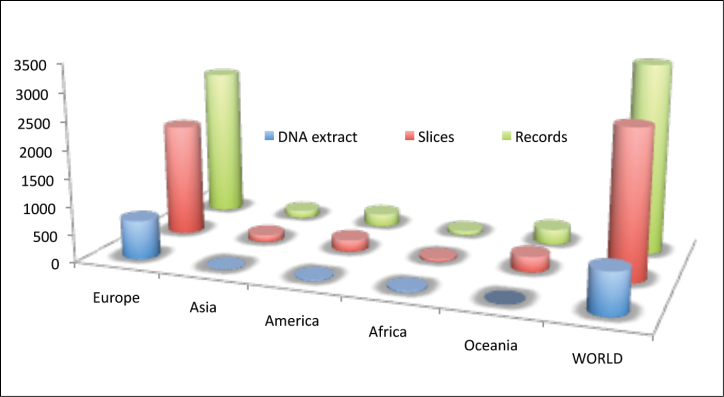
Number of records of Bathynellacea by continents in the MNCN collections.

**General spatial coverage**: Specimens from all around the world are included, from Alaska (USA) to New South Wales (Australia). Figure 12 includes the number of records per continent, as well as the part corresponding to permanent slices and DNA extracts. The material from the USA comes from a few samples collected in the states of Montana, Washington, Alaska and Texas, and some of the specimens are still pending identification. In total, the database has 200 records (19 corresponding to DNA extracts) from the four species of Bathynellidae and the two species of Parabathynellidae originating from the 18 localities visited in the previously mentioned states. There are also 25 records from three South American localities in Chile, Brazil and Argentina which represent three species in total. The Asian countries included in the collection are China, Thailand, Vietnam and a pair of localities from Mongolia and India, adding up to 149 records corresponding to six species from a total of nine localities. In the case of Africa, there are samples from Morocco (29 records, 12 DNA extracts, and two species in total from two localities) and Chad (41 records, 14 DNA extracts, and with a total of two species from a single locality). Australia is represented by samples from Queensland and New South Wales, adding to a total of 270 records from seven localities that include 13 species in total (some still undetermined).

**Figure 13.**
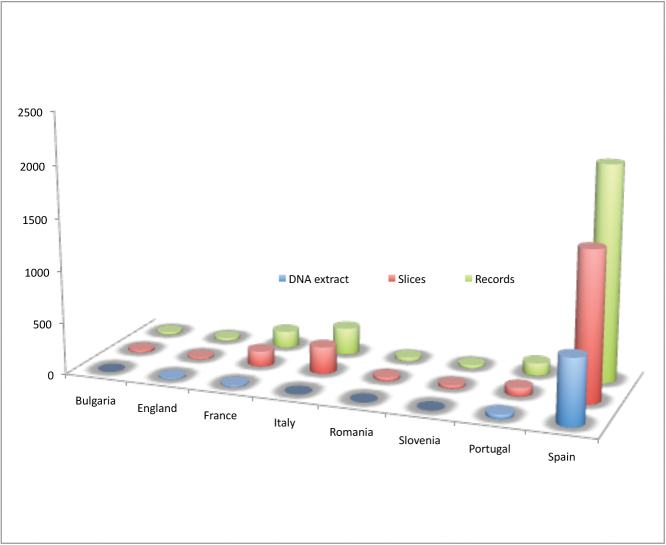
Number of records of Bathynellacea from Europe by countries in the MNCN collections.

The most important part of the database is composed by European records, especially from Spain (2064 records, including more than 50 species, with 631 DNA extracts), although other countries are also represented: Italy (256 records, 40 localities and 15 species), France (158 records, 12 DNA extracts, from 24 localities, and 12 species), Portugal (116 records, 38 DNA extracts, five localities and 11 species), England (28 records, 11 DNA extracts, four localities and a single species), Bulgaria (21 records, from three localities and four species), Slovenia (26 records, four localities and two species) and Romania (34 records, seven localities and six species) (Figure 13).

**Figure 14.**
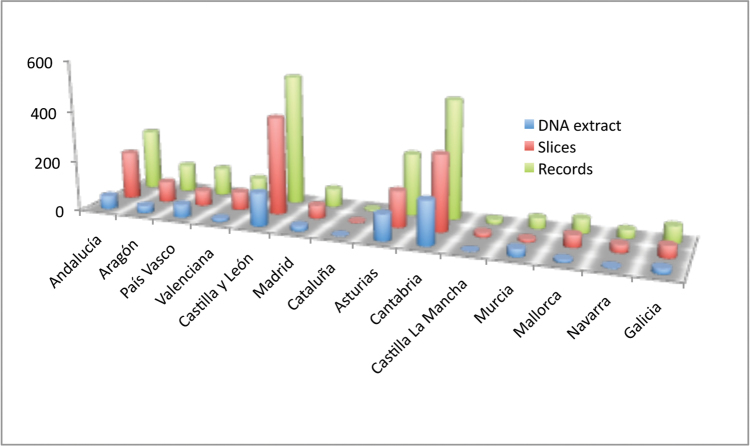
Number of records of Bathynellacea from Spain by Autonomous Communities in the MNCN collections.

In the case of Spain, almost all Autonomous Communities are represented (Figure 14), as well as most of the provinces, although Cantabria (472 records) and Burgos (373 records) are the most widely represented, followed by Asturias (245 records) and Soria, Vizcaya, Huesca and Teruel with more than 100 records for each province. There are records for seven of the eight Andalusian provinces (239 records in total): 76 records for Huelva, 57 for Sevilla, Málaga with 41 records, Almería with 35 records, Córdoba 18 records, Granada with nine records and Jaén with only three records. Cádiz is the only Andalusian province without any information in the database. Madrid has 71 records, Galicia 66, the Balearic Islands (only Mallorca) 57, Navarra 33 records and Catalonia with only four records. The rest of the provinces have relatively few records: León 24, Salamanca only 1, Guadalajara 14 records, Ávila and Toledo, both with four records. The only Autonomous Communities not present in the data base are Extremadura and La Rioja (Table 5).

**Table 5.** Records of Spanish Bathynellacea from Autonomous Communities and provinces in the collection of the MNCN. * Written in Spanish to keep the original name.

*Autonomous Communities	*Provinces	Records
Andalucía	Almería	35
	Cádiz	0
	Córdoba	18
	Granada	9
	Huelva	76
	Jaén	3
	Málaga	41
	Sevilla	57
Aragón	Huesca	>100
	Teruel	>100
	Zaragoza	0
Asturias	Asturias	**245**
Canarias	Las Palmas	0
	Santa Cruz de Tenerife	0
Cantabria	Cantabria	**472**
Castilla La Mancha	Albacete	0
	Ciudad Real	0
	Cuenca	0
	Guadalajara	14
	Toledo	4
Castilla y León	Ávila	4
	Burgos	**373**
	León	24
	Palencia	0
	Salamanca	1
	Segovia	1
	Soria	>100
	Valladolid	0
	Zamora	0
Cataluña	Barcelona	0
	Gerona	1
	Lérida	3
	Tarragona	0
Ciudades Autónomas	Ceuta	0
	Melilla	0
Comunidad de Madrid	Madrid	71
Comunidad Foral de Navarra	Navarra	33
Comunidad Valenciana	Alicante	75
	Castellón	3
	Valencia	23
Extremadura	Badajoz	0
	Cáceres	0
Galicia	La Coruña	0
	Lugo	44
	Orense	12
	Pontevedra	13
Islas Baleares	Baleares	57
La Rioja	La Rioja	0
País Vasco	Álava	14
	Guipúzcoa	0
	Vizcaya	>100
Región de Murcia	Murcia	44

There are 631 DNA specimens coming from basically all provinces, with the exception of Salamanca, Toledo and Jaén. Again the highest number of these specimens come from Cantabria (172 DNA extracts), followed by Asturias (142 DNA extracts) and Burgos (83 extracts). A detailed analysis of the distribution of species and localities where bathynellaceans live in Spain is available in a data paper previously published (Camacho et al., 2014).

### Coordinates

Latitude/longitude 62.323016/-148.014001 to -24.75764/152.38247

### Temporal coverage (specimens’ data range)

1968–2016

### Temporal coverage (collection formation)

1983–present

### Natural collections description

**Parent collection identifier**: NA

**Collection name**: Camacho Collection (AIC), Arthropods Collection and Tissues and DNA Collection

**Specimen preservation method**: permanent slices (glycerin jelly and paraffin) and frozen DNA extracts in water.

**Curatorial unit**: 3399 with an uncertainty of 0 (records)

## Methods

**Method step description**: The collection has been digitized with MSEXCEL software, compatible with Darwin Core 1.2 or Darwin Core 1.4.

*Pre-digitization phase*: The identifications of each specimen from each sample has been reviewed recently and some former imprecisions and the discovery of cryptic species (due for example to the use of molecular techniques) have lead modifying some records in the Excel file used as starting point for this work. The initial files were short on the number of fields for each of the records, specimens, sampling sites and dates of sampling (date, locality, province, habitat, collector and the species found with data on the family genus, species and author).

*Digitization phase*: Starting from the initial Excel file, the standard fields for a Darwin Corev1.2 database were added as needed, and the geographical data was included (UTM coordinates) from a GPS in association to the samples taken (PASCALIS samples and all those taken after the year 2000), or were obtained from grey (speleological reports) or published (Notenboom and Meijers 1984; Puch 1998) literature (i.e., the precise location through GPS in the entrance of the caves where bathynellid samples have been collected), or were recorded by the researchers who donated the specimens when possible, as well as from type specimens.

*Creation of the dataset*: The dataset was exported as a file in Darwin Core1.2 format. Darwin Core elements included in dataset structure are listed in the dataset description section. A Darwin Core table was prepared from the original database project. The field-to-filed mapping was fine-tuned with the support of GBIF-Spain’s Coordination Unit. The resulted table was imported into the Darwin Test tool (http://www.gbif.es/darwin_test/Darwin_test_in.php, Ortega-Maqueda and Pando, 2008). This tool allows detailed structuring of metadata of the dataset, and also performs a number of quality checks on the data (dataset structure compliance to Darwin core, geographic consistency, date format, etc. currently over sixty of those checks are carried out). Once the potential errors flagged have been checked and corrected, a Darwin Core Archive is generated, also by the DarwinTest tool. The produced DwC-A is then uploaded to the GBIF-Spain’s IPT installation (http://www.gbif.es/ipt/resource?r=mncn-artp). From there, the dataset is made public, registered in GBIF and indexed and published by the GBIF data portal.

The dataset was transformed to a Darwin Core Archive format with metadata to ensure rapid discovery of this biodiversity resource and future publishing as a citable academic paper (Chavan and Penev, 2011)

**Study extent description**: The MNCN bathynellacean collection begins with the sampling campaigns of AIC in northern Spain for her doctoral thesis since 1983. Some samples studied by AIC were obtained between 1976 and 1978 by R. Rouch in three short sampling trips to different areas of the Iberian Peninsula. From 1984 to 1986 J. Notenboom, assisted by I. Meijers, and later P. van der Hurk & R. Leys, took groundwater samples throughout Spain and all Bathynellacea they found in these samples were also donated to AIC for study. The following years AIC has continued obtaining samples of this fauna throughout Spain in the framework of different research projects. It is worth noting the PASCALIS European project (2002-2004) in which AIC and her team conducted intensive sampling of groundwater fauna in the Cantabrian mountain ranges and north of Burgos, an area where continuous sampling has been done since then, together with C. Puch, increasing substantially the number of Bathynellacea records in Spain. Occasional samplings of particular Parabathynellidae species have been done by AIC and C. Puch in touristic Spanish caves in Andalusia, Murcia and Galicia in order to obtain DNA extracts. On top of this, since the beginning of the 2000s, AIC has been receiving donations for her research coming from Spain, but also from other parts of the world (France, Italy, Bulgaria, England, USA, China, Vietnam, Thailand, Mongolia, Chad and Australia).

**Sampling description**: Material of this collection has been collected in five ways:

1) Samples collected by Rouch in two short sampling campaigns in the Iberian Peninsula (1976 and 1977).

2) Samples collected in the sampling campaigns of Notenboom, in 1984, 1985 and 1986 in the Iberian Peninsula within the framework of his PhD thesis.

3) Samples collected by AIC in 1983 for her PhD thesis (1987), plus samplings done in the framework of several research projects already mentioned, always with the collaboration of C. Puch and other speleologists (F. Molinero, A.M. de Juan, J. Robador, F. Lázaro, J. Bedoya) from 1984 until today.

4) Samples collected by AIC and her team as Spanish partners of the European Project “PASCALIS” (Cantabrian mountain range) (2002-2004).

5) Some particular samples, with more or less extensive associated information, have been donated to AIC by fellow researchers worldwide: E. Ortiz, D. Jaume, A. Tinaut, J. Rodríguez, A. García-Valdecasas, P. Rodríguez, E. Bello, C. Noreña, P. Martínez-Arbizu, J. Comas, L. Barrera, F. Mezquita, C. Prieto, E. Serban, N. Coineau, C. Boutin, C. Bou, J. Mathieu, M-J. Dole-Olivier, F. Castellerini, C. des Chatelliers, E. Castella, F. Malard, F. Stoch, D. Galassi, T. di Lorenzo, M.C. Bruno, B. Sket, P. Trontelj, P. Leclerc, Y. Ranga Reddy, M. Peralta, I. Pandoursky, S. Watiroyram, R. Newell, E. Snyder, J. Stanford, B. Reid, B. Hutchins, Gibson, J. Little, Z. Crete, P. Hancock and L. Knight.

The methods used in collecting this kind of samples can be seen in Camacho, 1992 and 1994. The samples are fixed in the field in formalin 4%, ethanol 96º, or are frozen. Each sample collected is studied under a binocular microscope in order to isolate the bathynellid specimens found.

The specimens used for morphological study are stored in alcohol (70%). The specimens used for molecular study are frozen at -80ºC. A complete dissection, of all anatomical parts of specimens, dropped on pure glycerin, is necessary for taxonomic study. Both, entire specimens or all parts of a dissection specimen are preserved together in permanent slides and kept in special metal slides. Glycerin gelatin stained with methylene blue and paraffin is the mounting medium (Figure 7). Anatomical examinations are performed using an oil immersion lens (100X) of an interference microscope. Method modified after Serban’s method personally transmitted to AIC in 1993 and 1995 (Perina and Camacho, 2016).

The specific techniques used for molecular analysis for taxonomic application are detailed in Camacho et al. 2011, 2012, 2013a, 2015 and 2016.

**Quality control description**: Systematics reliability and consistency is backed by the experience of AIC, who made all identifications in the field of Bathynellacea taxonomy. Recently, some identifications made are being confirmed by molecular data. The validation and cleaning of the associated geographical information has been introduced in several steps as a key issue of the digitization process.

## Datasets

### Dataset description

**Object name**: Darwin Core Archive The collection of Bathynellacea specimens of MNCN (CSIC) Madrid: microscope slices and DNA extracts.

**Character encoding**: UTF-8

**Format name**: Darwin Core Archive format

**Format version**: 1.2

**Distribution**: http://www.gbif.es/ipt/resource?r=mncn-artp

**Publication date of data**: 2016/11/22

**Update police**: Annually when necessary to transmit data of new samples or taxonomic changes.

**Language**: English

**Licenses of use**: This dataset [The collection of Bathynellacea specimens of MNCN (CSIC) Madrid: microscope slices and DNA extracts] is made available under the Open Database License: http://opendatacommons.org/licenses/odbl/1.0/. Any rights in individual contents of the database are licensed under the Database Contents License: http://opendatacommons.org/licenses/dbcl/1.0/.

**Metadata language**: English

**Date of metadata creation**: 2016/11/22

**Hierarchy level**: Dataset

## Contributions

The main collectors are J. Notenboom & I. Meijers, R. Rouch, A.I. Camacho (AIC) especially C. Puch and speleologist F. Molinero and A.M. de Juan, J. Robador and F. Lázaro members of G.E. Edelweiss, plus some particular donations by other Spanish researchers: E. Ortiz, D. Jaume, A. Tinaut, J. Rodríguez, A. García-Valdecasas, P. Rodríguez, E. Bello, C. Noreña, P. Martínez-Arbizu, J. Comas, L. Barrera, F. Mezquita and C. Prieto and other foreign researchers: E. Serban, N. Coineau, C. Boutin, C. Bou, L. Knight, J. Mathieu, M-J. Dole-Olivier, F. Castellerini, C. des Chatelliers, E. Castella, F. Malard, F. Stoch, D. Galassi, T. di Lorenzo, M.C. Bruno, B. Sket, P. Trontelj, P. Leclerc, Y. Ranga Reddy, M. Peralta, I. Pandoursky, S. Watiroyram, R. Newell, E. Snyder, J. Stanford, B. Reid, B. Hutchins, Gibson, J. Little, Z. Crete, P. Hancock and L. Knight.

## Online at

http://www.gbif.es/ipt/resource?r=mncn-artp

http://www.gbif.org/dataset/07f0789f-c777-4c99-acb3-815c78c7db81

http://doi.org/10.15470/t1lssy
